# Good results after treatment of  RAMP lesions in association with ACL reconstruction: a systematic review

**DOI:** 10.1007/s00167-022-07067-3

**Published:** 2022-07-23

**Authors:** Riccardo D’Ambrosi, Amit Meena, Akshya Raj, Riccardo Giorgino, Nicola Ursino, Laura Mangiavini, Jon Karlsson

**Affiliations:** 1 IRCCS Istituto Ortopedico Galeazzi, Via Galeazzi 4, 20161 Milan, Italy; 2 Dipartimento di Scienze Biomediche per la Salute, Università degli Studi di Milano, Via Mangiagalli 31, Milan, Italy; 3 Gelenkpunkt-Sports and Joint Surgery, Innsbruck, Austria; 4 Central Institute of Orthopaedics, Vardhman Mahavir Medical College and Safdarjung Hospital, New Delhi, India; 5 Department of Orthopaedics, Institute of Clinical Sciences, Sahlgrenska Academy, University of Gothenburg, Gothenburg, Sweden

**Keywords:** RAMP lesion, Medial meniscus injury, ACL reconstruction, Meniscal repair, Meniscectomy, Posterior horn

## Abstract

**Purpose:**

This study aimed to systematically evaluate the clinical, functional, and radiological outcomes, complications, and rate of return to sports among patients with RAMP lesion of the medial meniscus encountered during anterior cruciate ligament (ACL) reconstruction.

**Methods:**

A systematic review was conducted based on the PRISMA guidelines. Two independent reviewers searched the PubMed, Scopus, Embase, and Cochrane Library databases using the terms “ACL” or “anterior cruciate ligament,” and “RAMP lesion.” The outcome measures extracted from the studies were the Short Form-12 (SF-12) in its mental and physical component (MCS and PCS), Lysholm score, Subjective IKDC, Marx Score, WOMAC Score, Tegner, Radiological changes, complications, failures and/or revision surgery, and rate of return to sports.

**Results:**

The cohort of patients consisted of 1,243 participants with a mean age of 28.6 ± 2.6. The mean postoperative follow-up was 40.9 ± 6.3 months. A total of 1145 (92.1%) RAMP lesions were repaired with concomitant ACL reconstruction, while only 98 (7.9%) lesions were left untreated (or treated with abrasion only). The Lysholm score was used in 6 studies (in one only at final follow-up), with a significant improvement in all the studies (Lysholm_pre_ 60.03 ± 6.12; Lysholm_post_ 89.9 ± 5.0). Eight studies out of nine reported Subjective IKDC score, and a significant improvement was noted in all cases (IKDC_pre_ 56.2 ± 5.8. IKDC_post_ 84.9 ± 3.7). Of 18 (1.4%) complications reported, 15 (1.2%) were related to RAMP/ACL surgery, and of the remaining three (0.2%) two (0.2%) were hematomas and one (0.1%) a contralateral ACL lesion. Of the 106 (8.5%) revision surgeries required, 5 (0.4%) were in non-treated lesions [two (0.2%) ACL re-ruptures and three (0.2%) medial meniscus re-injury]. In treated patients, the revision occurred for the following reasons: 75 (6.0%) meniscectomy, 14 (1.1%) meniscal suture revisions, 11 (0.9%) ACL failures and one (0.1%) arthrolysis.

**Conclusions:**

It is not yet clear if, in all cases of ACL reconstruction in which a medial meniscal RAMP lesion is encountered, the lesion needs to undergo surgical repair. Accordingly, it is recommended that in the repair of all unstable medial meniscal RAMP lesions during an ACL reconstruction in cases associated with a stable RAMP lesion, the surgeon may decide on repair based on the patient profile.

**Level of evidence:**

Level IV.

**Supplementary Information:**

The online version contains supplementary material available at 10.1007/s00167-022-07067-3.

## Introduction

RAMP lesions are identified as a specific type of injury involving the peripheral attachment of the posterior horn of the medial meniscus, and more precisely they are caused by a peripheral vertical longitudinal detachment of the posterior horn of the medial meniscus due to tears of the meniscocapsular ligament, leading to meniscocapsular or meniscotibial separation [4, 7, 14, 27].

RAMP lesions are increasingly gaining attention in the orthopaedic field, especially due to their association with an anterior cruciate ligament (ACL) injury [7], with a significantly variable prevalence in concomitant ACL injury [5]. Their importance also lies in the fact that this injury is associated with increased anterior translation of the tibia, dynamic rotational laxity, and excessive rotational mobility of the knee [19, 20]. For these reasons, despite being found in a vascularized area, currently the recommended treatment appears to suggest surgical repair [2, 6, 8], even if a possible spontaneous healing has also been reported [17].

To clarify the treatment of RAMP lesions, several classifications have been proposed that divide them into subtypes [12, 21, 25]. It is also important to pay attention to possible risk factors, recently identified, such as bone contusion on the posterior medial tibial plateau, chronic injury, steeper tibial and medial meniscal slope, gradual lateral tibial slope, and varus knee alignment > 3° [16].

Despite the recent increased awareness, RAMP injuries remain significantly underdiagnosed, for instance due to the use of the classic anterior arthroscopic portal, which limits the complete visualization of the posterior horn of the medial meniscus and its meniscocapsular junction, and therefore the visualization of RAMP lesions or the presence of a membrane-like tissue that might hide the aforementioned lesions, their being made visible only after a certain degree of debridement through a posteromedial portal [23].

Moreover, there is low RAMP injury detection on preoperative magnetic resonance imaging (MRI). It may be useful to look for indirect signs such as a bruise of the posteromedial tibial bone that has been found to be a secondary sign of a RAMP injury [6, 9].

Undoubtedly, this topic deserves greater attention to clarify the diagnostic–therapeutic algorithm in the face of these complexly managed lesions.

This study aimed to systematically evaluate the clinical, functional, and radiological outcomes, complications, and rate of return to sports among patients with RAMP lesion of the medial meniscus encountered during anterior cruciate ligament (ACL) reconstruction.

## Materials and methods

The current systematic review was performed following the Preferred Reporting Items for Systematic Reviews and Meta-Analyses (PRISMA) guidelines and is registered in the PROSPERO Registry (CRD42022335486) [18].

### Eligibility criteria

The literature selected for this study was based on the following criteria.

#### Study design

Randomized controlled trials (RCTs), controlled (non-randomized) clinical trials (CCTs), prospective and retrospective comparative cohort studies, case–control studies, and case series were included. Case reports and case series that did not report data on clinical and functional results were excluded.

#### Participants

Studies conducted on skeletally mature patients treated for RAMP lesion of the medial meniscus in association with anterior cruciate ligament (ACL) reconstruction. and were evaluated through a minimum follow-up of 1 year were considered eligible.

#### Interventions

Studies that reported data on clinical, functional, and radiological outcomes following the ACL reconstruction associated with RAMP lesion of the medial meniscus, independently if treated surgically or conservatively.

For ACL reconstruction the surgical technique (type of graft used, numbers of bundles, fixation technique, and tensioning protocol), and rehabilitation protocol were collected as well as approach and surgical technique for meniscal RAMP repair.

#### Types of outcome measures

The outcome measures extracted from the studies were the Short Form-12 (SF-12) in its mental and physical component (MCS and PCS), Lysholm score, Subjective IKDC, Marx Score, WOMAC Score, Tegner, radiological changes, complications, failures and/or revision surgery, and rate of return to sports.

RAMP lesions were classified according to the current literature as follows:

1. Thaunat et al. [25] approached the tear pattern, direction, thickness (partial vs. full), and associated meniscocapsular disruption, peripheral zone, or meniscotibial ligament lesion and instability (Type 1: meniscocapsular tear; Type 2: partial superior tear; Type 3: partial inferior tear; Type 4: complete tear; Type 5: double tear)
2. Greif et al. [12] in an extended Thaunat classification version integrate the recent knowledge from cadaveric studies showing that meniscocapsular and meniscotibial ligaments merge in their posterior horn meniscal attachment (Type 1: meniscocapsular ligament tear; Type 2: partial superior peripheral posterior meniscal horn tear; Type 3A: partial inferior peripheral posterior horn meniscal tear; Type 3B: meniscotibial ligament tear; Type 4A: complete peripheral posterior horn meniscal tear; Type 4B: complete meniscocapsular junction tear; Type 5: peripheral posterior horn meniscal double tear)
3. Seil et al. [21] approached the mediolateral extent of tears, degree of capsular attachment injury, and adherent (self-heal) vs. dehiscent (repair).

### Information sources and search

A systematic search for relevant literature was performed on the PubMed (MEDLINE), Scopus, EMBASE, and Cochrane Library databases. The publication date was not considered an inclusion criterion. The search was carried out in April 2022. Two independent reviewers (RD and AM) assisted in conducting and validating the search. The following search terms were entered in the title, abstract, and keywords fields: “ACL” or “anterior cruciate ligament,” and “RAMP lesion.” Lastly, only papers published in English were included.

### Data collection and analysis

#### Study selection

The retrieved articles were first screened by title and, if found relevant, screened further by reading the abstract. After excluding studies not meeting the eligibility criteria, the entire content of the remaining articles was evaluated for eligibility. To minimize the risk of bias, the authors reviewed and discussed all the selected articles, references, as well as the articles excluded from the study. In case of any disagreement between the reviewers, the senior investigator made the final decision. At the end of the process, further studies that might have been missed were manually searched by going through the reference lists of the included studies and relevant systematic reviews.

#### Data collection process

The data was extracted from the selected articles by the first two authors using a computerized tool created with Microsoft Access (Version 2010, Microsoft Corp, Redmond Washington). Every article was validated again by the first author before analysis. For each study, data regarding the patients was extracted (age, gender, duration between injury and surgery, and follow-up evaluation), their injuries (type, aetiology, and associated injuries), the surgical technique (type of graft used, numbers of bundles, fixation technique, and tensioning protocol), rehabilitation protocol, post-operative outcomes, rate of complications, and the rate of return to sports.

#### Level of evidence

The Oxford Levels of Evidence set by the Oxford Centre for Evidence-Based Medicine was used to categorize the level of evidence [11].

#### Evaluation of the quality of studies

The quality of the selected studies was evaluated using the Methodological Index for Nonrandomized Studies (MINORS) score [22]. The checklist includes 12 items, of which the last four are specific to comparative studies. Each item was given a score of 0–2 points. The ideal score was set at 16 points for non-comparative studies and 24 for comparative studies.

## Results

### Search results

**Fig. 1:**
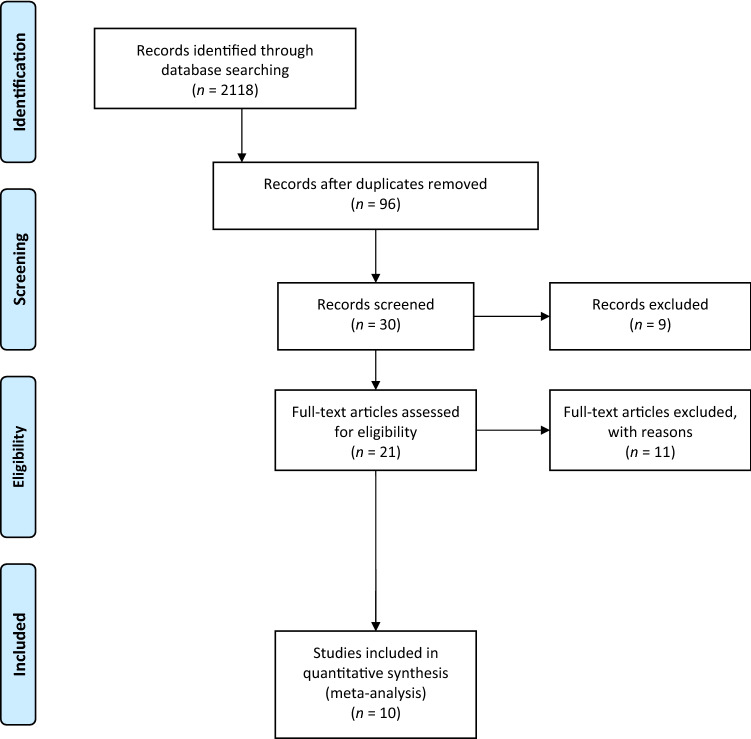
A flowchart of the literature screening performed in this study

The electronic search yielded 2118 studies. After 2022 duplicates were removed, 96 studies remained, of which 66 were excluded after reviewing the abstracts, bringing the number down to 30. An additional 20 articles were excluded based on the aforementioned inclusion and exclusion criteria. No additional studies were found by manually searching the reference lists of the selected articles. This left 10 studies for analysis [1, 3, 8, 10, 13, 15, 17, 24, 26, 28]. Figure 1 shows the flowchart depicting the selection process for studies. The analyzed studies had a mean MINORS score of 12.9 (range, 9–18), which confirmed the methodological quality of the available literature (Table 1).

**Table 1:** Characteristics of the selected studies

Authors, Year	MINORS	Level of Evidence	Patients (*n*)	M: F *(n*)	Age Mean ± SD (range)	Time between injury and surgery	FU	Ramp lesion classification	Aetiology/ mechanism of injury (*n*)	Associated injuries (*n*)
Albayrak 2020 [1]	9	III	33	32:1	28.3 ± 7.8	15.8 weeks	47.3 ± 9.4 months	Type 1	25 contact injury 8 non-contact	n.a
Balzas 2020 [3]	11	III	67	36:31	28.4	n.a	1–5 years	36 Stable 31 unstable	23 contact 44 non-contact	n.a
Chen 2017 [8]	12	IV	46	34:12	26 (18–41)	n.a	32 (14–36) months	18 Meniscotibial ligament tear (MLT) 13 meniscocapsular tear (MCT), 15 combined MLT/MCT	n.a	n.a
DePhilippo 2020 [10]	18	III	50	22:28	30.5 ± 11.4	31 acute 19 chronic	2.8 (2–8) years	n.a	n.a	n.a
Hatayama 2020 [13]	18	III	50 25 non repaired 25 repaired	Non repaired: 13:12 Repaired: 17:8	Non repaired: 29.5 Repaired: 26.6	Non repaired: 680 days Repaired: 494	24 months	25 Stable 25 Unstable	n.a	n.a
Liu 2017 [17]	18	II	73 40 repaired 33 abraded and trephined	n.a	Repaired: 35.6 ± 8.5 Abraded and trephined: 34.8 ± 9.1	Repaired: 9.4 ± 12.1 Abraded and trephined: 8.3 ± 10.5	Repaired: 37.9. ± 15.9 Abraded and trephined: 40.3 ± 16.5	n.a	n.a	n.a
Sonnery-Cottet 2018 [24]	9	IV	416	n.a	n.a	n.a	45.6 (24–2 – 66.2) months	n.a	n.a	n.a
Thaunat 2022 [26]	11	III	248	174:74	29.5 ± 9.5	107 < 3 months 141 > 3 months	46.4 ± 9.6 months (31–72)	Type 1 129 Type 2 13 Type 3 27 Type 4 64 Type 5 15	n.a	n.a
Thaunat 2016 [28]	11	IV	132	110:22	26.4 (12–57)	n.a	27 (24–29)	n.a	n.a	45 lateral meniscal tear
Keyhani 2016 [15]	12	IV	128	107:21	24 (18–48)	n.a	24 (24–47)	n.a	n.a	n.a

*n.a*.  not available; *FU*  follow-up; *MINORS* Methodological Index for Non-Randomized Studies

### Patient and study characteristics

Table 1 shows the characteristics of the cohorts involved in the 10 selected studies and a summary of their data. The cohort of patients consisted of 1,243 participants (545 (43.8%) men and 209 (16.8%) women – 2 studies did not report ratio M:F [17, 24]) with a mean age of 28.6 ± 2.6 (range 12–57). The mean postoperative follow-up was 40.9 ± 6.2 months (range, 14–72 months). Five studies [1, 3, 8, 13, 26] reported RAMP classification and were divided as follows: 162 (13.0%) type 1, 13 (1.0%) type 2, 27 (2.2%) type 3, 64 (5.1%) type 4, 15 (1.2%) type 5, 61 (4.9%) stable, 56 (4.5%) unstable, 18 (1.44%) meniscotibial ligament tear (MLT), 13 (1.0%) meniscocapsular tear (MCT), 15 (1.2%) combined MLT/MCT.

### Origin

Only 2 studies [1, 3] reported type of injuries, and in 48 (3.9%) cases there was a sports contact injury while 52 (4.2%) cases had a non-sports contact injury.

### Surgical protocol

#### ACL

All data in terms of the surgical technique followed in each of the examined studies are displayed in “Appendix”. All studies reported the type of graft used, except two [3, 15]. Only in one study was the use of a double bundle technique reported [13].

### RAMP

#### Repaired

All studies except one [1] reported RAMP lesion repair with different techniques as reported in “Appendix”, for a total of 1145 (92.1%) lesions.

#### Unrepaired

In one study [1] only were all RAMP lesions not treated, while in the study of Balzas et al. [3] 32 (2.6%) stable RAMP lesions were not treated and in the study of Liu et al. [17] 33 (2.6%) RAMP lesions were treated with abrasion and trephination for a total of 98 (7.9%) lesions.

#### Rehabilitation protocol

Only three studies reported the use of postoperative brace [1, 8, 17]; partial weight bearing was granted from day 0 in 2 studies [10, 24], while in remaining studies it ranged from 2nd to 4th week post the operation. For range of motion all studies reported different protocol ranging from early range of motion after discharge to complete full extension for 4 weeks post-operative.

#### Clinical and functional outcomes

Two studies reported clinical evaluation using SF-12, and Balzas et al. [3] found no difference among different treatments. Alabaryak et al. [1] noted significant improvement between pre- and post-operative PCS and MCS SF-12 (MCS_pre_ 53.0 ± 1.35; MCS_post_: 55.8 ± 2.9; PCS_pre_ 43.8 ± 3.3 PCS_post_ 54.2 ± 0.6) [1, 3].

Lysholm score was used in 6 studies (in one only at final follow-up), with a significant improvement reported in all the papers (Lysholm_pre_ 60.0 ± 6.1; Lysholm_post_ 89.9 ± 5.0) [1, 8, 10, 13, 15, 17].

Eight studies out of ten reported Subjective IKDC score, and in all cases a significant improvement was noted (IKDC_pre_ 56.2 ± 5.8. IKDC_post_ 84.9 ± 3.7) [1, 3, 8, 10, 15, 17, 26, 28].

Marx score was used only by Balzas et al., with no differences between the different treatment groups (*p *> 0.05) [3]; WOMAC score was reported only by DePhilippo, with a significant improvement (*p* < 0.05) [10], whereas Tegner was reported in three studies (of which one only was post-operative), with contrasting results (Tegner_pre_ 5.8 ± 2.3; Tegner_post:_ 7.0 ± 0.5) [10, 13, 28].

#### Post-operative changing

At second look arthroscopy, Chen et al. noted complete healing in 40 (3.2%) cases, incomplete healing in 5 (0.4%), and in 1 (0.08%) failure after repair using the FastFix System [8].

Hatayama et al. reported complete healing in 10 (0.8%) cases, partial healing in five (0.4%), and 10 (0.8%) unhealed non-repaired lesions, while in repaired lesions 20 (1.6%) healed and 5 (0.4%) partially healed were noted on MRI [13].

In his randomized clinical trial, Liu et al. observed 38 (3.1%) healed, one (0.1%) partially healed, and one (0.1%) non-healed in sutured lesions, while the abrasion group reported 29 (2.3%) healed, two (0.2%) partially healed, and two (0.2%) non-healed [17].

Thaunat et al. reported 12 (1.0%) non-healed on post-operative MRI [28].

Detailed results are reported in Table 2.

**Table 2:** Clinical and functional outcomes, complications, and return to sports and activity

Lead Author	SF-12 MCS		SF-12 PCS		Lysholm		Subjective IKDC		Marx		Womac total		Tegner		Changing	Complications	Failures/Revisions	Return to sport
	Pre	Post	Pre	Post	Pre	Post	Pre	Post	Pre	Post	Pre	Post	Pre	Post				
Albayrak 2020 [1]	53.6 ± 2.6	58.5 ± 2.4*	41.7 ± 2.7	54.8 ± 2.6*	54.1 ± 6.5	86.0 ± 6.4*	44.4 ± 7.8	77.4 ± 9.2*								4: 1 Movement limitations 1 acl tear in opposite knee 2 pain related to the implants	0	28 Patients returned to sports: 3 lower level 25 same level
Balzas 2020 [3]	Stable untreated: 51.3 (9.4) Unstable ramp— partial meniscectomy: 52.8 (6.7) Unstable ramp— Repaired: 53.6 (9.2)	Stable untreated: 51.7 (8.6) Unstable ramp— partial meniscectomy: 57.8 (3.4) Unstable ramp— Repaired: 55.9 (3.2)	Stable untreated: 41.8 (7.3) Unstable ramp— partial meniscectomy: 49.8 (8.3) Unstable ramp— Repaired: 43.9 (9.5)	Stable untreated: 54.3 (4.5) Unstable ramp— partial meniscectomy: 53.1 (7.2) Unstable ramp— Repaired: 54.2 (6.1)			Stable untreated: 49.5 (15.5) Unstable ramp— partial meniscectomy 64.0 (10.4) Unstable ramp— Repaired: 50.7 (12.8)	Stable untreated: 85.6 (10.8) Unstable ramp— partial meniscectomy: 87.7 (13.1) Unstable ramp— Repaired: 80.6 (17.2)	Stable untreated: 12.8 (4.2) Unstable ramp— partial meniscectomy: 9.0 (5.2) Unstable ramp— Repaired: 11.9 (5.8)	Stable untreated: 9.3 (5.0) Unstable ramp— partial meniscectomy: 8.3 (4.2) Unstable ramp— Repaired: 9.3 (5.7)							Stable untreated: 2 ACL re-rupture 1 medial meniscus re-injury Unstable ramp— partial meniscectomy: 1 medial meniscus re-injury Unstable ramp— Repaired: 2 ACL re-rupture 5 medial meniscus re-injury	
Chen 2017 [8]					56.81 (37–70)	94.44 (90–99)*	52.74 (28–66)	90.59 (80–98)*							Second Look Arthroscopy: 40 complete healing 5 incomplete healing 1 failed	2 mild articular cartilage of MFC 1 implant displaced	5 partial meniscectomy revision 1 repeated repair revision 3 rasp refresh revision 1 ACL revsion	
DePhilippo 2020 [10]					53 [31, 69]	86 [80, 95]*	66 [62, 72]	78 [72, 80]*			28 [17, 52]	0 [0, 8]*	2 [1, 3]	8 [6, 9]*		1 cyclops lesion 1 ORIF patellar fracture 2 arthrofibrosis 1 MCL injury	1 medial meniscus revision repair 1 partial meniscectomy revision	84% returned to their preinjury activity
Hatayama 2020 [13]						Non repaired: 98.5 Repaired: 98.7								Non repaired: 6.0 Repaired: 6.8	MRI: non repaired 10 completely healed 5 partially healed 10 unhealed Repaired 20 completely healed 5 partially healed		Non repaired: 2 subsequent meniscecomy	
Liu 2017 [17]					Sutured: 68.6 ± 6.1 abrasion: 64.3 ± 7.5	Sutured: 88.7 ± 4.8* Abrasion: 90.4 ± 5.8*	Sutured: 51.1 ± 5.5 Abrasion: 53.6 ± 6.7	Sutured: 83.6 ± 3.7* Abrasion: 82.2 ± 4.5*							MRI: Sutured: Completely healed 38 Partially healed 1 Nonhealed 1 Abrasion: Completely healed 29 Partially healed 2 Nonhealed 2			
Sonnery-Cottet 2018 [24]																	45 partial meniscectomy	
Thaunat 2022 [26]							56 ± 12	88 ± 10*									15 meniscectomy 3 sutures 8 acl failures 4 cyclops syndrome 1 arthrolysis	
Thaunat 2016 [28]							63.8 ± 13.5	85.7 ± 12*					7.2 ± 1.92	6.9 ± 1.72	MRI. 12 unhealed	2 hematoma	9 meniscal revision	82% of patients returned to their premorbidity activity
Keyhani 2016 [15]					61.7 ± 3.2	87.8 ± 3.9*	53.6 ± 2.1	82.1 ± 3.5*									3 meniscectomy	

*SF*-12 Short Form-12; *MCS* Mental component; *PCS* Physical component; *IKDC* The international knee documentation committee; *WOMAC* Western Ontario and McMaster university; *MRI* Magnetic resonance imaging

Statistically significant versus pre-operative score (*p* < 0.05)

#### Return to sports

Only three studies analyzed return to sports, and in all these studies more than 80% of the patients returned to their pre-injury activity [1, 10, 28].

#### Complications and revisions surgery

Of 18 (1.4%) complications reported, 15 (1.2%) were related to RAMP/ACL surgery (one (0.1%) had movement limitations and two (0.2%) were due to arthrofibrosis, in two (0.2%) pain related to the implants, one (0.1%) had implant displacement, five (0.4%) had cyclops lesion, one (0.1%) a patellar fracture, one (0.1%) MCL injury, two (0.2%) MFC cartilage lesion), and of the remaining three (0.2%) two (0.2%) were hematomas and one (0.1%) a contralateral ACL lesion.

Of the 106 (8.5%) revision surgeries required, 5 (0.4%) were in non-treated lesions (two (0.2%) ACL re-ruptures and three (0.2%) medial meniscus re-injury). In treated patients the revision occurred for the following reasons: 75 (6.0%) meniscectomy, 14 (1.1%) meniscal suture revisions, 11 (0.9%) ACL failures, one (0.1%) arthrolysis. Detailed results are reported in Table 2.

## Discussion

The most important findings of this analysis were that the most commonly reported outcome score in the studies included in the study, the subjective IKDC score, showed significant functional improvement for all the treatment methods used for RAMP lesion repair along with ACL reconstruction, while the second most commonly reported outcome score in the included studies, the Lysholm score, also showed significant functional improvement in all the studies it was reported in.

The Tegner activity score, which was reported in three of the included studies, showed improvement, although this was not statistically significant. Therefore, there is clinically significant functional improvement in knees where ACL reconstruction was performed along with RAMP lesion repair.

In the study by Albayrak et al., non-treatment of stable unrepaired RAMP lesions with ACL reconstruction did not show lower functional knee scores than isolated ACL reconstructions [1],

whereas the study by Balzas et al. showed no significant difference in outcomes between non-repaired medial meniscal root lesions and those repaired with ACL reconstruction surgery [3].

Three studies had analyzed return to sports activity at a preinjury level, namely the studies by Dephilippo et al., Albayrak et al., and Thaunat et al. [1, 10, 28]. The study by Thaunat et al. [28] found an 82% rate of return to preinjury level of activity. Moreover, the study by Albayrak et al. [1] reported no significant differences in return to sports rates between isolated ACL reconstruction and ACL reconstruction with a non-repaired stable RAMP lesion, while that of Dephilippo et al. [10] found no significant differences between patients who underwent ACL reconstruction with a meniscal RAMP repair and patients of isolated ACL reconstruction, in return to sports activity. Further, the study by Balzas et al. [3] found no significant differences between ACL reconstructions with concomitant non-repaired stable RAMP lesions and those with a repaired unstable RAMP lesion. Therefore, one may suggest that despite the presence of a RAMP lesion, repairing these lesions may not be necessary while undertaking a concomitant ACL reconstruction, if the RAMP lesion is found to be stable intraoperatively, at least in most patients. However, returning to sports at the same activity level took a significantly longer period for the group with RAMP lesions than for those with isolated ACL reconstructions, in the study by Albayrak et al. [1]. As such, it may be worthwhile, in high demand populations such as professional sportspersons, to repair a stable RAMP lesion, which may ensure earlier return to preinjury levels of sports activity participation. The unstable meniscal RAMP lesion needs to be repaired for healing of the lesion and subsequent good knee function.

Healing of RAMP lesions was not significantly different with respect to repair and abrasion-trephination, as reported by Liu et al. [17]. The healing rate of RAMP lesions was significantly higher in the repaired group compared with unrepaired lesions on postoperative MRI in the study by Hatayama et al. [13], who reported good healing rates (87%). Hence, surgical repair of RAMP lesions appears to ensure good rates of healing. A low rate of complications was found upon review of all the studies. The rate of failure and overall rate of revision repair was also low in the articles reviewed. The included studies showed a low rate of conversion to partial meniscectomy due to failure and reinjury.

To the best of our knowledge, this is the first systematic review in terms of ACL reconstruction with concomitant RAMP lesions of the posterior horn of the medial meniscus. However, by the very nature of a systematic review, the collection of data is limited to the studies available in the literature and what those studies report. Limitations of the current systematic review are a lack of studies with higher level of evidence in the literature, with only a few studies having been done on ACL reconstruction with a concomitant RAMP lesion, the fact that there is only one prospective randomized controlled study, the heterogenous nature of the techniques used to treat the RAMP lesion, and non-uniform reporting of outcome scores across the studies.

## Conclusion

With the currently available data, it is not yet clear if all cases of ACL reconstruction in which a medial meniscal RAMP lesion is encountered should undergo repair of the lesion. With that said, repair of RAMP lesion appears to hasten the return to sporting activity, without much impact on the overall rate of return to sports. Accordingly, we recommend the repair of all unstable medial meniscal RAMP lesions during an ACL reconstruction, while in cases associated with a stable RAMP lesion the surgeon may decide on repair based on the patient profile. Randomized prospective studies with greater size of study populations will need to be undertaken in order to make more concrete recommendations regarding the management of ACL injury with associated RAMP lesions.

### Electronic supplementary material

Below is the link to the electronic supplementary material.

Supplementary file 1 (DOCX 81 KB)

## Appendix

See Table 3 below.

**Table 3:** Surgical and rehabilitation protocols

Lead Author	Graft type	Fixation technique		Surgical technique	Ramp lesion treatment	Bundle	Tension protocol	Rehabilitation protocol		
		Femur	Tibia					Brace/Splint	Full weight-bearing	ROM time
Albayrak 2020 [1]	Four-strand hamstring	n.a	n.a	n.a	Untreated	Single bundle	n.a	Hinged knee brace for 4 weeks	4 weeks	90º was not allowed until the end of the third week
Balzas 2020 [3]	n.a	n.a	n.a	n.a	32 stable—untreated 23 unstable ramp repaired with all inside technique 12 unstable ramp—meniscectomy	n.a	n.a	n.a	n.a	n.a
Chen 2017 [8]	Hamstring	Bioresorbable IS + staples	Endobutton	n.a	Repaired using FastFix System	Single Bundle	n.a	Hinged brace	6 weeks	0 to 90 degrees at 4 weeks
DePhilippo 2020 [10]	BTPB	Cannulated titanium IS	Cannulated titanium IS	Anatomic	Inside-out suture	Single Bundle	Full extension	No	Weight as tolerated upon discharge	24 h after surgery initiate early range of motion
Hatayama 2020 [13]	Semitendinosus tendon	Endobutton	2 staples	Anatomic	All-inside by posteromedial portal	Double bundle	15°	No	3 weeks	1 week after surgery
Liu 2017 [17]	Four-strand hamstring	n.a	n.a	n.a	40: From posteromedial portal with suture hook 33: abrasion and trephination without surgical repair	Single bundle	n.a	Hinged Brace	Partial weightbearing at 2 weeks; full weightbearing at 4 weeks	Full extension for 4 weeks
Sonnery-Cottet 2018 [24]	Quadrupled semitendinosus tendons, bone–patellar tendon–bone,1quadrupled hamstring tendons, or in the case of combined ACL–anterolateral ligament grafts, a tripled semitendinosus with a single strand of gracilis	n.a	n.a	n.a	From posteromedial portal with suture hook	n.a	n.a	no	Weightbearing as tolerated from day 0	0–90° for the first 4 weeks
Thaunat 2022 [26]	16 BPTB 232 Hamstring + ALLR	n.a	n.a	n.a	38 All-inside device 184 suture hook repair 16 all-inside + suture hook 10 missing data	n.a	n.a	n.a	n.a	n.a
Thaunat 2016 [28]	89 hamstring 41 BPTB 2 quadriceps	n.a	n.a	n.a	All-inside posterior Lasso sutures; all-inside fast-fix; out-in	n.a	n.a	no	From week 3	0–90° for 6 weeks
Keyhani 2016 [15]	n.a	n.a	n.a	Anatomic	Posteromedial portal with suture hook	n.a	n.a	no	Toe-touch weight for 2 weeks, partial weight-bearing 2–4 weeks	Passive 0–45° for 2 weeks 0–90° 2–4 weeks

*BPTPB* Bone-patellar tendon-bone; *ALLR* Anterolateral ligament reconstruction; *IFS* Interference screw; *ROM* Range of motion
