# Supplementary material for: Good results after treatment of RAMP lesions in association with ACL reconstruction: a systematic review
Source: Knee Surg Sports Traumatol Arthrosc. 2022 Jul 23;31(1):358–71. doi: 10.1007/s00167-022-07067-3 (PMC9859864; doi:10.1007/s00167-022-07067-3)
Supplement: Supplementary file 1 — (DOCX 81 KB) [file 167_2022_7067_MOESM1_ESM.docx]

**Table 2.** Surgical and Rehabilitation Protocola

|  |  | Fixation technique | |  |  |  |  | Rehabilitation protocol | | |
| --- | --- | --- | --- | --- | --- | --- | --- | --- | --- | --- |
| Lead Author | Graft Type | Femur | Tibia | Surgical Technique | Ramp Lesion Treatment | Bundle | Tension protocol | Brace / Splint | Full weight-bearing | ROM time |
| Albayrak 2020 [1] | Four-strand hamstring | n.a. | n.a. | n.a. | Untreated | Single bundle | n.a. | Hinged knee brace for 4 weeks | 4 weeks | 90º was not allowed until the end of the third week |
| Balzas 2020 [3] | n.a | n.a. | n.a. | n.a. | 32 stable - untreated  23 unstable ramp repaired with all inside technique  12 unstable ramp –meniscectomy | n.a. | n.a. | n.a. | n.a. | n.a. |
| Chen 2017 [8] | Hamstring | Bioreasorbable IS + staples | Endobutton | n.a. | Repaired using FastFix System | Single Bundle | n.a. | Hinged brace | 6 weeks | 0 to 90 degrees  at 4 weeks |
| DePhilippo 2020 [10] | BTPB | Cannulated titanium IS | Cannulated titanium IS | Anatomic | Inside-out suture | Single Bundle | Full extension | No | weight as tolerated upon discharge | 24 hours after surgery initiate early range of  motion |
| Hatayama 2020 [13] | Semitendinosus tendon | Endobutton | 2 staples | Anatomic | All-inside by posteromedial portal | Double bundle | 15° | No | 3 weeks | 1 week after surgery |
| Liu 2017 [17] | Four-strand hamstring | n.a. | n.a. | n.a. | 40: From posteromedial portal with suture hook  33: abrasion  and trephination without surgical repair | Single bundle | n.a. | Hinged Brace | Partial weightbearing at 2 weeks  Full wightbearing at 4 weeks | Full extension for 4 weeks |
| Sonnery-Cottet 2018 [24] | Quadrupled semitendinosus  tendons, bone–patellar tendon–bone,1quadrupled  hamstring tendons, or in the case of combined ACL–  anterolateral ligament grafts, a tripled semitendinosus  with a single strand of gracilis | n.a. | n.a. | n.a. | From posteromedial portal with suture hook | n.a. | n.a. | no | Weightbearing as tollerated from day 0 | 0-90° for the first 4 weeks |
| Thaunat 2022 [26] | 16 BPTB  232 hamstring + ALLR | n.a. | n.a. | n.a. | 38 all-inside device  184 suture hook repair  16 all-inside + suture hook  10 missing data | n.a | n.a. | n.a. | n.a. | n.a. |
| Thaunat 2016 [28] | 89 hamstring  41 BPTB  2 quadriceps | n.a. | n.a. | n.a. | All-inside posterior Lasso sutures;  all-inside fast-fix; out-in | n.a. | n.a. | no | From week 3 | 0-90° for 6 weeks |
| Keyhani 2016 [15] | n.a | n.a. | n.a. | Anatomic | Posteromedial portal with suture hook | n.a. | n.a. | no | Toe-touche weight for 2 weeks, partial weight-bearin 2-4 weeks | Passive 0-45° for 2 weeks  0-90° 2-4 weeks |

BPTPB=Bone-Patellar Tendon-Bone; ALLR=anterolateral ligament reconstruction; IFS, interference screw; ROM=range of motion.

a
